# A Systematic Literature Review with Meta-Analyses of Within- and Between-Day Differences in Objectively Measured Physical Activity in School-Aged Children

**DOI:** 10.1007/s40279-014-0215-5

**Published:** 2014-07-01

**Authors:** Hannah L. Brooke, Kirsten Corder, Andrew J. Atkin, Esther M. F. van Sluijs

**Affiliations:** UKCRC Centre for Diet and Activity Research (CEDAR) and MRC Epidemiology Unit, University of Cambridge School of Clinical Medicine, Box 285 Institute of Metabolic Science, Cambridge Biomedical Campus, Cambridge, CB2 0QQ UK

## Abstract

**Background:**

Targeting specific time periods of the day or week may enhance physical activity (PA) interventions in youth. The most prudent time segments to target are currently unclear.

**Objectives:**

To systematically review the literature describing differences in young people’s objectively measured PA on weekdays vs. weekends, in school vs. out of school, weekends vs. out of school and lesson time vs. break time.

**Methods:**

Electronic databases were searched for English-language, cross-sectional studies of school-aged children (4–18 years) reporting time-segment-specific accelerometer-measured PA from 01/1990 to 01/2013. We meta-analysed standardised mean differences (SMD) between time segments for mean accelerometer counts per minute (TPA) and minutes in moderate-to-vigorous PA (MVPA). SMD is reported in units of standard deviation; 0.2, 0.5 and 0.8 represent small, moderate and large effects. Heterogeneity was explored using meta-regression (potential effect modifiers: age, sex and study setting).

**Results:**

Of the 54 included studies, 37 were eligible for meta-analyses. Children were more active on weekdays than weekends [pooled SMD (95 % CI) TPA 0.14 (0.08; 0.20), MVPA 0.42 (0.35; 0.49)]. On school days, TPA was lower in school than out of school; however, marginally more MVPA was accumulated in school [TPA −0.24 (−0.40; −0.08), MVPA 0.17 (−0.03; 0.38)]. TPA was slightly lower on weekends than out of school on school days, but a greater absolute volume of MVPA was performed on weekends [TPA −0.10 (−0.19; −0.01), MVPA 1.02 (0.82; 1.23)]. Heterogeneity between studies was high (*I* ^2^ 73.3–96.3 %), with 20.3–53.1 % of variance between studies attributable to potential moderating factors.

**Conclusions:**

School-aged children are more active on weekdays than weekend days. The outcome measure influences the conclusions for other comparisons. Findings support the tailoring of intervention strategies to specific time periods.

**Electronic supplementary material:**

The online version of this article (doi:10.1007/s40279-014-0215-5) contains supplementary material, which is available to authorized users.

## Key Points

**Table:** 

Notable differences in physical activity were observed between specific time periods of the day and the week, with school-aged children generally more active on weekdays than weekend days
Time-segment comparisons, other than weekdays compared with weekend days, were influenced by whether the unit of physical activity measurement was absolute (i.e. minutes spent in moderate-to-vigorous intensity physical activity) or relative (i.e. mean accelerometer counts per minute)
The findings suggest that there may be greater scope to influence physical activity during some time segments of the day or week than others, and therefore support the tailoring of intervention strategies to specific periods of time

## Introduction

Physical activity is important for health in children and adolescents; it has been inversely associated with metabolic syndrome [1] and clustered cardiovascular risk factors [2–4], and positively associated with bone health [5]. It is recommended that children accumulate 60 min of moderate-to-vigorous intensity physical activity (MVPA) each day [6], although many do not achieve this [7, 8]. In addition, physical activity declines substantially throughout adolescence [9, 10]. To date, interventions to promote physical activity in young people have had limited success in changing whole-day physical activity [11], but there is some evidence that interventions delivered during a specific period of the day may be beneficial. For example, an overall positive effect has been observed for after-school interventions [12], and several reviews support the effectiveness of interventions during school time [13]. However, the impact of these interventions on whole-day physical activity is unclear. Further research informing intervention design is necessary to overcome the public health challenges associated with insufficient physical activity.

Identifying correlates and determinants of behaviour is an important stage in the sequence of research steps leading to the development of evidence-based interventions [14]. A range of correlates and determinants of physical activity in young people have been studied [15], but temporal factors have received limited attention. While many studies descriptively report physical activity for specific time segments of the day and week, few conduct formal statistical tests of differences in activity between time segments. Previous reviews have compared time-segment-specific physical activity across studies or have limited comparisons of weekdays versus weekends [16–19]. Interventions often target particular times of the day, such as school [13, 20], afterschool [12, 21] or recess [22]. However, justification for their target time segment is largely based on pragmatic arguments, such as the ability to recruit a whole school or the possibility to utilise resources and facilities. An alternative approach would be to target times of the day or week which offer the greatest scope to influence physical activity. Considered in conjunction with the contribution of each time segment to children’s overall activity, identifying specific times of the day or week when physical activity is particularly low might indicate time segments during which intervention could be most beneficial.

Objective measures are increasingly being used to study physical activity levels in children and adolescents [23]. Objective data facilitate the investigation of temporal correlates of physical activity by allowing detailed quantification of physical activity throughout the day and the week. Objective measures eliminate recall bias [24] and provide a more precise measurement of physical activity. These benefits lead to more accurate estimates of effect sizes, the ability to monitor children consistently across time, and the possibility of performing comparisons between studies [25].

We aimed to systematically review and meta-analyse published literature summarising time-segment-specific differences in objectively measured physical activity in healthy school-aged children. This research will inform intervention targets and provide direction for further research into the time-based influences on physical activity in young people.

## Methods

### Search Strategy and Screening Protocol

A protocol for this review was agreed by all co-authors before commencing literature searches. Four electronic databases—(1) MEDLINE via PubMed, (2) Scopus, (3) Science Citation Index (SCI) via Web of Knowledge, and (4) SPORTDiscus via EBSCOhost—were searched from January 1990 until January 2013. The search start date was based on preliminary scoping work which identified the emergence of relevant research. Searches were within title, abstract and keywords for all databases except MEDLINE, for which title and abstract only were searched. Four groups of search terms, based on the themes of young people, physical activity, objective measures, and time segments, were combined using Boolean operators (see Table S1 of the “Electronic Supplementary Material”, ESM). No language limitations were enforced.

**Fig. 1:**
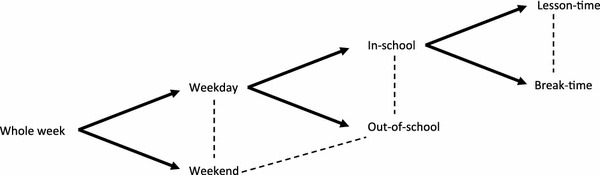
A hierarchical model illustrating time segments of the day and week. *Dashed lines* indicate comparisons of physical activity between specific time periods of the day and the week evaluated in the current study

The initial screening of full texts identified many papers (*n* = 235) which met the inclusion criteria of (1) a study sample of school-aged children from nonclinical populations, (2) an objective measure of physical activity, and (3) physical activity data reported for two or more time segments. Additional criteria were developed to refine the focus of the review. First, physical activity was most commonly measured using accelerometers (*n* = 155 papers). To reduce heterogeneity between outcomes, all papers reporting other objective measures of physical activity were excluded. Second, a heterogeneous set of time-segment comparisons were identified. The time-segment comparisons illustrated in Fig. 1 were hypothesised to be most informative for intervention design. Each additional level of comparison contributed more detailed information about physical activity levels across the day and throughout the week. Articles only presenting other comparisons were therefore excluded.

**Fig. 2:**
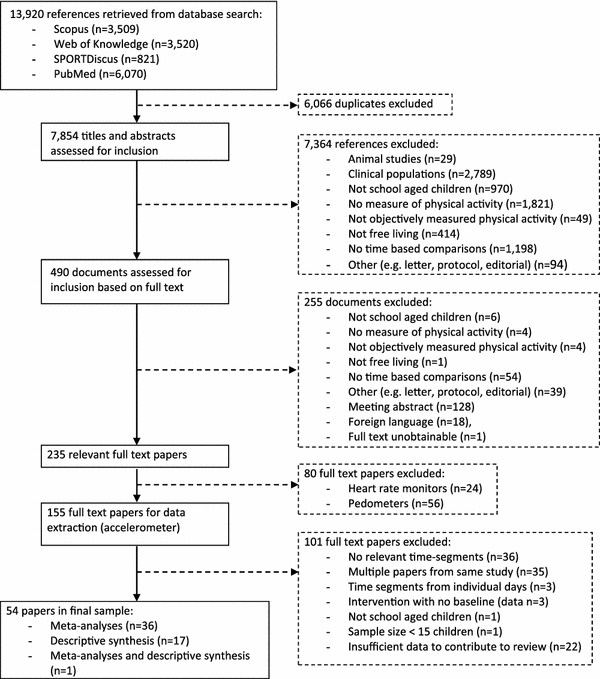
Flow chart of the article selection process

The article selection process is described in Fig. 2. Following the removal of duplicates, the titles and abstracts of all articles were screened by the first author (HLB). Potentially relevant articles were retrieved for full text screening. Where full texts were not available online, study authors were contacted and a copy of their paper was requested. HLB screened all full texts and conducted data extraction for papers in the final sample.

### Inclusion Criteria

To be included in this review, articles must have fulfilled the following criteria:

- Study sample of school-aged children (4–18 years) recruited from a nonclinical population. Children recruited from preschool settings were excluded as their daily patterns of physical activity are likely to vary substantially from school-aged children.
- Physical activity reported for specific time segments: weekdays and weekend days and/or in school and out of school and/or weekends and out of school and/or lesson time and break time (Fig. 1).
- Study outcome of physical activity measured by accelerometer (all manufacturers/models included).
- Studies of cross-sectional observational design or longitudinal and intervention studies (providing baseline data) that were published in a peer-reviewed journal. For longitudinal studies with a baseline measurement during preschool, the first data collection in school years was taken as the baseline.

In addition, we only included available full-papers written in English at the full text screening stage. Abstracts from meetings and conferences were excluded as full texts were necessary to extract methodological details. Multiple papers from the same study presenting the same comparison(s) were excluded to prevent overrepresentation. Papers were retained according to a pre-specified priority order of (1) largest sample size and (2) earliest publication date.

### Data Extraction

Studies that presented a mean and standard deviation of total physical activity (TPA), reported as counts per minute (CPM), and/or time spent in MVPA, reported in minutes, were eligible for meta-analyses. Data were synthesised for TPA and MVPA separately for each time-segment comparison. More than three suitable studies were required for each meta-analysis. Data that were only presented as figures in the original paper were not included in meta-analyses. Relevant studies that were unsuitable for meta-analysis but that presented a relevant statistically tested comparison were included in a descriptive data summary.

For studies suitable for meta-analysis, a standardised data extraction form was used to extract the mean and standard deviation of TPA and/or MVPA in each time segment as well as study-level data on descriptive characteristics and potential moderators. Data extraction was conducted at subgroup level (e.g. sex or age group), which were subsequently treated as independent samples. In papers with multiple subgroup stratifications, a pre-specified priority order of country, sex, age, weight status, socioeconomic status, ethnicity, transport mode and “other” was used to assess inclusion. For studies included in the descriptive summary, the main results and outcomes of statistical tests were extracted alongside study level data on descriptive characteristics.

### Statistical Analyses

Standardised mean differences (Cohen’s* d*) in physical activity between time segments (weekdays vs. weekend days, in school vs. out of school, weekends vs. out of school and lesson time vs. break time) were calculated for each study [26]. Data for each time-segment comparison were then combined through a random effects meta-analysis using the *metan* command in Stata (StataCorp, release 12 (2011), Stata Statistical Software, College Station, TX, USA). To assess variation in standardised mean difference attributable to heterogeneity, the *I* ^2^ statistic was inspected. If the *I* ^2^ statistic suggested substantial heterogeneity (i.e. *I* ^2^ > 50 %) [26], this was investigated using meta-regression. We tested the association of each potential effect modifier (age, sex and global region of study setting) with the standardised mean difference in physical activity between time segments for each comparison. Age was taken as the mean age presented in each study; if mean age was not reported, the median value based on the reported range was used. Sex was separated into three categories: boys, girls or both (the final category was for studies which did not present data separately for boys and girls). A categorical variable was created for global region of study setting; studies were classified as Europe, North America or other. Significantly associated factors (*p* < 0.05) were combined into a multivariate model to calculate the overall variance between studies that could be explained.

It is conceivable that physical activity is correlated between different time segments of the day and the week. However, as correlations between time segments were largely unreported, it was not possible to account for them in the main analyses; therefore, a correlation of zero between time segments was assumed for all studies. Sensitivity analyses were conducted to assess the effect of potential correlation by assuming high (*r* = 0.8), medium (*r* = 0.5) and low (*r* = 0.2) correlations between time segments in all studies [26, 27]. Two post hoc sensitivity analyses were conducted. The first tested the weekday vs. weekend day comparison and excluded studies that reported different criteria on weekdays and weekend days for the minimum number of minutes of registered activity required for a day to be included (*n* = 9). The second excluded studies that reported after-school activity for the time period immediately after school only (for example between 3 pm and 5 pm) or which did not report the time that “after school” started and ended (*n* = 4).

## Results

Of the 7,854 documents identified in the original search, 54 were included in the final sample. Thirty-six studies were suitable for meta-analysis. Between 26 and 63 independent samples were included in meta-analyses. A further 17 studies were summarised descriptively, and one study was included in both descriptive and meta-analytic syntheses (Fig. 2). The descriptive characteristics of the studies included in meta-analyses and descriptively synthesised studies are presented in Table 1; detailed study-by-study characteristics are presented in Table S2 of the ESM.

**Table 1:** Descriptive characteristics of the included studies, divided into those which were meta-analysed and those which were descriptively synthesised

	Studies included in meta-analyses		Descriptively synthesised studies^a^	
	*n* reporting characteristic (/36)	*n*	*n* reporting characteristic (/18)	*n*
Sample size (*n*)	36		18	
15–99		5		10
100–249		13		5
250–499		6		3
500–999		5		0
≥1,000		7		0
Sample age groups studied (years)^b^	36		18	
4–10		27		17
11–14		23		9
≥15		11		2
Global region	36		18	
Europe		23		10
North America		8		3
Australasia		3		1
Other		2		4
Ethnicity	11		2	
Majority white		6		2
Mixed (i.e. no one group >50 %)		3		0
Majority other than white		2		0
SES	21		3	
Low or low–medium		7		2
Medium–high		9		1
Other		5		0

*SES* Socioeconomic status

^a^Includes one study which reported data suitable for meta-analysis and data unsuitable for meta-analysis

^b^Studies could include children in more than one age group, so they could be counted multiple times

The pooled standardised mean differences indicate that children were more active on weekdays than weekends; however, the effect was weaker for TPA than MVPA (Table 2 and Fig. S1 in the ESM). On school days, children had lower TPA in school than out of school; however, marginally more minutes of MVPA were accumulated in school (Table 2 and Fig. S2 of the ESM). Similarly, TPA was slightly lower on weekends than out of school on school days, but a greater volume of MVPA was accumulated on weekends (Table 2; Fig. S3 in the ESM).

**Table 2:** Summary of physical activity reported in meta-analysed studies and corresponding results of meta-analyses

Outcome	Time-segment comparison	*n* independent samples	Mean ± SD (CPM or mins)	Range (CPM or mins)		Pooled SMD	95 % CI of pooled SMD		Interpretation of size of effect^a^	*I* ^2^ (%)^b^
				Min	Max		Lower	Upper		
TPA	Weekdays	52	600.1 ± 121.2	323.9	901.0	0.14	0.08	0.20	Small	83.5
	Weekend	52	569.3 ± 124.8	302.9	803.0	Ref				
MVPA	Weekdays	63	82.3 ± 44.0	18.8	200.6	0.42	0.35	0.49	Small-medium	86.1
	Weekend	63	68.3 ± 43.9	11.8	187.1	Ref				
TPA	In school	36	555.9 ± 191.6	150.0	954.0	−0.24	−0.40	−0.08	Small	94.8
	Out of school	36	596.7 ± 175.6	178.0	873.0	Ref				
MVPA	In school	29	34.4 ± 14.6	11.0	72.9	0.17	−0.03	0.38	Small	96.3
	Out of school	29	32.8 ± 17.1	10.2	69.0	Ref				
TPA	Weekend	26	596.5 ± 118.1	385.0	803.0	−0.10	−0.19	−0.01	Small	73.3
	Out of school	26	629.1 ± 119.6	431.0	873.0	Ref				
MVPA	Weekend	29	83.1 ± 38.6	34.0	168.6	1.02	0.82	1.23	Large	94.6
	Out of school	29	42.5 ± 17.7	18.0	95.1	Ref				

*TPA* total physical activity, *MVPA* moderate-to-vigorous intensity physical activity, *SD* standard deviation, *CPM* counts per minute, *Min* minimum, *Max* maximum, *SMD* standardised mean difference, *CI* confidence interval, *Ref* reference time segment

^a^Interpretation of size of effect: 0.2 was considered a small effect, 0.5 a moderate effect, and 0.8 a large effect [27]

^b^ *I* ^2^ indicates the proportion of the variability in effect estimates that is due to heterogeneity

Heterogeneity between studies was high (*I* ^2^ = 73.3–96.3 %) (Table 2). Meta-regression models revealed that the age, sex and global region of study setting were weakly associated with the standardised mean difference in physical activity between time segments for some comparisons (Tables S3a and S3b in the ESM). The direction and size of the effect varied with comparison and specific outcome of interest. Age was most consistently associated with the standardised mean difference in physical activity between time segments, whereas global region was only associated with the standardised mean difference in MVPA for the weekend vs. out of school comparison. Potential moderating factors explained 20.33–53.1 % of the variance between studies.

Sensitivity analyses showed that including a correlation between time segments in the meta-analyses did not alter the results (Table S4 in the ESM). The larger the assumed correlation between time segments, the smaller the confidence intervals around the effect estimates, so the results presented here (assuming a correlation of zero) are the most conservative estimate. Results were largely unchanged in sensitivity analyses that examined the impact of differential wear time criteria for weekdays versus weekend days or the use of shortened or unspecified after-school definitions.

A descriptive summary of studies that were unsuitable for meta-analysis but that reported statistically tested relevant comparisons is presented in Table 3. Half the studies that presented a comparison between weekdays and weekends indicated that children participated in more physical activity on weekdays [28–35]. Others indicated that there was no significant difference in physical activity between weekdays and weekends [36–40] or presented mixed results depending on characteristics of the sample such as age and sex [41] or characteristics of the analysis such as the intensity of physical activity examined [42]. There was inconclusive evidence for the comparison of in school vs. out of school. Only two studies statistically tested this comparison; one indicated that MVPA was similar in school and out of school [43]; the other indicated that MVPA was lower in school than out of school [44]. Only one eligible study presented break-time and lesson-time physical activity, and this showed that children had significantly higher TPA during break time than lesson time [45].

**Table 3:** Description of results from studies unsuitable for meta-analysis

Study	Year	Outcome	Units of outcome	Main result	*P* value
Weekdays–weekend					
Deforche et al. [28]	2009	TPA	CPM	Weekday > weekend	<0.001
Frömel et al. [29]	2012	TPA	Step/day	Weekday > weekend	<0.001
Frömel et al. [30]	2008	TPA	kcal kg^−1^ day^−1^	Weekday > weekend	<0.001
Godard et al. [31]	2012	TPA	CPM/hour	Weekday > weekend	<0.001
Rowlands et al. [32]	2008	TPA	Counts/day	Weekday > weekend	<0.05
Soric et al. [33]	2010	TPA	Minutes/day	Weekday > weekend	0.001
Stone et al. [34]	2009	TPA	Counts/day	Weekday > weekend	<0.01
Sherar et al. [35]	2009	MPA and VPA	Minutes/day	Weekday > weekend	<0.05
Basterfield et al. [36]	2011	TPA	Median CPM	Weekday = weekend	0.488
Jürisson et al. [37]	1996	TPA	kcal without resting metabolic rate	Weekday = weekend	>0.05
Wilkin et al. [38]	2006	TPA	Mean units × 10^5^/day	Weekday = weekend (in 2 age groups of children)	0.19 and 0.43 (boys) 0.14 and 0.21 (girls)
Kemp et al. [39]	2011	TPA and MVPA	kcal and % of whole day	Weekday = weekend	0.26 (TPA) 0.794 (MVPA)
McManus et al. [40]	2011	MPA and VPA	Minutes/day	Weekday = weekend	>0.05
Trost et al. [41]	2000	MVPA	Minutes/day	Mixed depending on age and sex	
Esliger et al. [42]	2010	MPA and VPA	Minutes/day	Mixed depending on urbanity and intensity	
In school to out of school					
Jáuregui et al. [43]	2011	MVPA	Minutes/hour	In school = out of school	0.1
Silva et al. [44]	2011	MVPA	Minutes/day	In school < out of school	0.018
Lesson time to break time					
Rush et al. [45]	2012	TPA	CPM	Lesson < break	<0.00001

*TPA* total physical activity, *MVPA* moderate-to-vigorous intensity physical activity, *VPA* vigorous intensity physical activity, *CPM* counts per minute

## Discussion

Meta-analyses of published cross-sectional data revealed notable differences in physical activity between specific periods of the day and week in young people. Results provide further support for the notion of tailoring physical activity interventions to specific periods of time, which has been shown previously to be a promising intervention strategy [12, 13].

Aside from the comparison between weekdays and weekend days, the results were influenced by whether the unit of physical activity measurement was absolute (i.e. minutes spent in MVPA) or relative (i.e. TPA, expressed as mean accelerometer CPM). As would be expected, the absolute measure was sensitive to time-segment duration, with more minutes of MVPA accumulated during longer time segments. The relative measure was more robust when comparing physical activity during time segments of differing durations. These findings emphasise that if absolute measures of physical activity are used, then it may be necessary to take into account measurement duration not only when comparing time segments within a study but also when drawing comparisons between studies. These differences also show that if an intervention successfully increases the average intensity of physical activity by a small amount in a time segment that is longer, the prolonged exposure may lead to large gains in terms of absolute activity accumulated. Furthermore, the discrepancies between absolute and relative measures highlight the importance of clarifying intervention aims before selecting a time segment to target. Physical activity guidelines in the United Kingdom recommend that children participate in 60 minutes of MVPA each day [6]. Some interventions may therefore aim to increase the absolute volume of MVPA participated in by young people. Others may be concerned with increasing CPM; for example, by encouraging light-intensity physical activity in place of sedentary behaviour. The subtle differences in these intervention aims may alter the most appropriate time segment to target or strategy to pursue. Interestingly, most of the included studies that reported both absolute and relative comparisons did not comment on the differences observed [46–51]. Nilsson et al. [48] touch on the issue and indicate that it was appropriate to present absolute values of MVPA in their study rather than MVPA relative to registered time; they suggest that different time segments provide different opportunities for physical activity. Given the increasing use of objective monitoring devices and interest in time-specific patterns of physical activity, the impact and implications of using relative versus absolute metrics should be considered carefully in future research.

### Weekdays vs. Weekend Days

Both MVPA and TPA were lower at weekends than on weekdays; the effect was small to medium when MVPA was the outcome (approximately 14 min per day) and small when TPA was used (approximately 31 CPM). Whilst these differences appear relatively small, 14 minutes of MVPA equates to almost 25 % of children’s recommended daily volume of physical activity [6]. Extrapolating data from a previous study in children shows an additional 31 CPM of daily total physical activity can be estimated to equate to about a 1 mm Hg lower diastolic blood pressure [52]. Although the clinical relevance of this for children is currently unclear, in adults a 2 mm Hg reduction in diastolic blood pressure has been estimated to result in a 6 % reduction in the risk of coronary heart disease events and a 15 % reduction in the risk of stroke and transient ischemic attacks [53]. Weekday and weekend time segments were likely to have been a similar length, which may account for the consistency of results observed for absolute and relative PA metrics. At weekends children have a greater choice in how they spend their time, and the results suggest that they generally do not choose to spend it participating in physical activity. Lower activity on weekends compared with weekdays was supported by some studies that were not suitable for meta-analysis. Despite heterogeneity across studies in the meta-analyses and the lack of association in some statistically tested comparisons, confidence in this finding has been strengthened by the relative consistency of evidence for both MVPA and TPA. Results are also consistent with studies that have assessed physical activity by pedometer and other objective methods [17–19].

### In School vs. Out of School

On weekdays, children accumulated more MVPA during school hours than outside of school, though the difference was small (approximately 2 min per day) and of borderline significance. Whilst much of the school day is spent in class, active play during break times and physical education provide potentially important opportunities for young people to engage in MVPA [54–58]. The presence of friends and the availability of relatively safe, open spaces in which to be active may also be important in this regard. Interestingly, the results obtained for TPA contrasted with those seen for MVPA, such that average activity intensity was greater out of school than during school hours. It may be hypothesised that whilst school affords the opportunity to engage in higher-intensity activity at specific times of the day, the predominant requirement to be seated during class time lowers average activity intensity when considering the school day as a whole. The duration of the out-of-school period varied across the included studies, with some studies focussing on a very short period immediately after school [46, 59]. Studies have shown a large peak in accelerometer counts per minute immediately before and after school, particularly in those using active modes of transport such as walking or cycling [60]. Therefore, school travel may be one factor responsible for increasing the average physical activity intensity during out-of-school time. In post hoc sensitivity analyses, the results for both MVPA and TPA were minimally affected if studies that focussed on the hours immediately after school were removed from the meta-analysis. This supports the idea that the nature of physical activity may differ in and out of school, rather than the difference being a result of school travel.

### Out of School vs. Weekend

Analysis revealed that children accumulated more MVPA during the weekend relative to the out-of-school hours on weekdays (approximate difference of 40 min per day). Given the shorter duration of the out-of-school period relative to weekend days, this is perhaps unsurprising. As was the case for the in-school versus out-of-school comparison, the direction of difference for TPA contrasted with that seen for MVPA; average activity intensity was slightly higher outside of school (approximately 33 CPM) than at the weekend. These contrasting findings likely reflect the different intensities and durations of activities undertaken during these periods, but it is currently unclear which specific behaviours may drive this observation. Future research that focuses in more detail on differences in activity patterns between specific time periods of the day and the week, perhaps combining behavioural assessment with information obtained from monitoring devices, may shed light on this issue and help to inform intervention design [61, 62].

### Heterogeneity

Substantial heterogeneity was observed in the meta-analyses, which was partially explained by potential effect modifiers studied in meta-regression. The results of the meta-regression analyses were interpreted cautiously due to the large number of tests that were run and the low consistency of the results. The difference between in-school vs. out-of-school TPA and weekend vs. out-of-school TPA and MVPA was smaller for older children. Therefore, interventions targeting a particular time segment may have scope for greater effect in some age groups of children than others. In contrast, the global region of the study setting was not a significant effect modifier for most comparisons. Heterogeneity in time-segment-specific physical activity has been reported between different countries [48]. It was therefore important to account for geographical location as a potential effect modifier. Studies included in meta-analyses were from 16 different countries, so they were grouped by global region. However, different climates, environments and physical activity cultures can exist in the same global region, this variability may have obscured differences. Other studies have indicated a seasonal variation in physical activity [63–65]. The season of data collection and related environmental factors such as the weather and hours of daylight were potential sources of heterogeneity that we were unable to assess in the current study. However, they may be important to explore in future studies as associations may differ over the course of the year. Methodological differences in accelerometer data, such as the type of monitor used, epoch length, MVPA count threshold, and the number of zero counts considered to indicate “non-wearing” of the accelerometer, may also have influenced the results [66] and contributed to heterogeneity. However, meta-analysing the standardised mean difference between time segments accounts for studies which measure the same outcome but in a number of different ways. In addition, the methodology within each study was consistent for all time segments examined. Therefore, factors related to accelerometer methodologies were not included as potential effect modifiers.

### Implications for Interventions and Future Research

Targeting weekend physical activity could be an important avenue for future interventions as both MVPA and TPA were lower at the weekend than on weekdays. Existing interventions have seldom targeted weekend physical activity specifically. The weekend may be of particular interest because previous research has shown that as children age, physical activity declines more during the weekend than on weekdays [67]. There are fewer constraints on young people’s time during the weekend, so there may be greater scope to implement an intervention. For interventions focussing on weekdays, it may be beneficial to target out-of-school hours since the least MVPA was accumulated in this time segment. There is evidence that after-school physical activity is predictive of overall physical activity, suggesting that there may be a synergistic effect of activity accumulated during these hours [68]. An alternative strategy could be to target time segments with lower TPA, such as in school and at the weekend, and aim to shift the intensity distribution in these time segments towards higher intensities.

To aid intervention development, the findings should be considered alongside research into time-specific correlates and determinants of physical activity [69–71]. This literature indicates that the most appropriate modifiable factors to target in interventions may differ between time segments. For example, family logistic support may be important for weekend physical activity, but peer support may be important for weekday physical activity [69]. In addition, age and sex are correlates of school break-time physical activity, and these factors plus body mass index, TV viewing/playing video games and access to facilities are correlates of physical activity in the after-school time period [71].

Studies included in the meta-analyses were largely conducted in economically developed countries, so additional research is required in less-developed countries. Due to the lack of data that is available for some comparisons, future research should investigate time segments other than weekdays and weekends, and should statistically test the differences between time segments. Breaking the day into smaller time segments could also be informative to further specify when to target physical activity interventions in young people. Few longitudinal studies examine changes in time-segment-specific physical activity from childhood into adolescence. These detailed studies are necessary to provide further information about prudent time segments to target in interventions focussing on physical activity maintenance.

### Strengths and Limitations

This study had a broad search strategy and included data from children and adolescents, a wide variety of countries, and a range of study designs. By focussing the review on studies with accelerometer-measured physical activity, we avoided the possibility of reporting bias and increased the confidence in the estimate of physical activity in comparison to self-report measures [24]. Reporting both relative and absolute measures of physical activity has been shown in this review to provide complementary information and highlight important differences which are not always considered when study outcomes are chosen or interventions designed. This study was the first, to our knowledge, to meta-analyse the differences in activity between time segments. Conducting meta-regression allowed sources of heterogeneity to be explored rather than just quantified.

Despite these strengths, we have also identified a number of limitations. Many accelerometers have limited ability to measure physical activity when children cycle or participate in water-based activities. Differential measurement bias may have been introduced if these activities were more prevalent in some time segments than others. Similarly, weekday and weekend day time segments theoretically have more equal durations than other time segments that were compared. Whilst some studies used different wear time criteria for week and weekend days [72–77], sensitivity analyses indicated that this did not impact on the results. We did not account for the possibility that in some countries children may attend school on Saturdays. Studies conducted in countries where school is attended on Saturdays may report more similar levels of physical activity on weekdays and weekend days than in other countries. This may result in more conservative effect sizes; however, we anticipate the impact of this on the results to be low. Relevant articles may have been overlooked if the title and abstract were not sufficiently detailed or if they were not indexed in the four searched databases. Moreover, non-English language papers were excluded and the findings cannot be generalised to countries beyond those included in the review. Many studies were excluded because data were not suitable for meta-analysis and comparisons had not been statistically tested; however, this methodology allowed more certain and quantified conclusions to be drawn. It is possible that publication bias affected the results, but it is neither desirable nor undesirable for children to be more active in one time segment than another, so it is unlikely that authors would systematically choose not to publish certain results.

## Conclusions

School-aged children are generally more active on weekdays compared to weekend days, but the comparison of other time segments is influenced by the outcome measure applied. The findings support the notion of tailoring physical activity interventions towards specific periods of time, but the best time segment to target depends on whether the intervention is aiming to increase volume of MVPA or increase average TPA.

## Electronic supplementary material

Below is the link to the electronic supplementary material.

Supplementary material 1 (PDF 217 kb)

## References

[1] Brage S, Wedderkopp N, Ekelund U (2004). Features of the metabolic syndrome are associated with objectively measured physical activity and fitness in Danish children: the European Youth Heart Study (EYHS). Diabet Care.

[2] Andersen LB, Harro M, Sardinha LB (2006). Physical activity and clustered cardiovascular risk in children: a cross-sectional study (The European Youth Heart Study). Lancet.

[3] Jimenez-Pavon D, Konstabel K, Bergman P, et al. Physical activity and clustered cardiovascular disease risk factors in young children: a cross-sectional study (the IDEFICS study). BMC Med. 2013;11. doi:10.1186/1741-7015-11-172. 10.1186/1741-7015-11-172 PMC3728104 23899208

[4] Ekelund U, Luan J, Sherar LB (2012). Moderate to vigorous physical activity and sedentary time and cardiometabolic risk factors in children and adolescents. JAMA.

[5] Ondrak KS, Morgan DW (2007). Physical activity, calcium intake and bone health in children and adolescents. Sports Med.

[6] Department of Health, Physical Activity, Health Improvement and Protection. Start active, stay active: a report on physical activity from the four home countries’ Chief Medical Officers. 2011. http://www.dh.gov.uk/prod_consum_dh/groups/dh_digitalassets/documents/digitalasset/dh_128210.pdf.

[7] Ekelund U, Tomkinson GR, Armstrong N (2011). What proportion of youth are physically active? Measurement issues, levels and recent time trends. Br J Sports Med.

[8] Hallal PC, Andersen LB, Bull FC, et al. Global physical activity levels: surveillance progress, pitfalls, and prospects. Lancet. 2012;380(9838). doi:10.1016/s0140-6736(12)60646-1. 10.1016/S0140-6736(12)60646-1 22818937

[9] Nader PR, Bradley RH, Houts RM (2008). Moderate-to-vigorous physical activity from ages 9 to 15 years. JAMA..

[10] Dumith SC, Gigante DP, Domingues MR (2011). Physical activity change during adolescence: a systematic review and a pooled analysis. Int J Epidemiol.

[11] Metcalf B, Henley W, Wilkin T. Effectiveness of intervention on physical activity of children: systematic review and meta-analysis of controlled trials with objectively measured outcomes (EarlyBird 54). Br Med J. 2012;345. doi:10.1136/bmj.e5888. 10.1136/bmj.e5888 23044984

[12] Beets MW, Beighle A, Erwin HE, et al. After-school program impact on physical activity and fitness: a meta-analysis. Am J Prev Med. 2009;36(6):527–37. doi:10.1016/j.amepre.2009.01.033. 10.1016/j.amepre.2009.01.033 19362799

[13] Kriemler S, Meyer U, Martin E, et al. Effect of school-based interventions on physical activity and fitness in children and adolescents: a review of reviews and systematic update. Br J Sports Med. 2011;45(11). doi:10.1136/bjsports-2011-090186. 10.1136/bjsports-2011-090186 PMC3841814 21836176

[14] Sallis JF, Owen N (1999). Physical activity and behavioral medicine.

[15] Craggs C, Corder K, van Sluijs EMF, Griffin SJ. Determinants of change in physical activity in children and adolescents: a systematic review. Am J Prev Med. 2011;40(6). doi:10.1016/j.amepre.2011.02.025. 10.1016/j.amepre.2011.02.025 PMC3100507 21565658

[16] Tudor-Locke C, McClain JJ, Hart TL (2009). Expected values for pedometer-determined physical activity in youth. Res Q Exerc Sport.

[17] Clemes SA, Biddle SJH (2013). The use of pedometers for monitoring physical activity in children and adolescents: measurement considerations. J Phys Act Health.

[18] Beets MW, Bornstein D, Beighle A (2010). Pedometer-measured physical activity patterns of youth. A 13-country review. Am J Prev Med.

[19] Armstrong N, Welsman JR (2006). The physical activity patterns of European youth with reference to methods of assessment. Sports Med.

[20] Dobbins M, Husson H, DeCorby K, et al. School-based physical activity programs for promoting physical activity and fitness in children and adolescents aged 6 to 18. Cochrane Database Syst Rev. 2013(2). doi:10.1002/14651858.CD007651.pub2. 10.1002/14651858.CD007651.pub2 PMC7197501 23450577

[21] Pate RR, O’Neill JR (2009). After-school interventions to increase physical activity among youth. Br J Sports Med.

[22] Parrish A-M, Okely AD, Stanley RM (2013). The effect of school recess interventions on physical activity: a systematic review. Sports Med.

[23] Rowlands AV (2007). Accelerometer assessment of physical activity in children: an update. Ped Exerc Sci.

[24] Reilly JJ, Penpraze V, Hislop J (2008). Objective measurement of physical activity and sedentary behaviour: review with new data. Arch Dis Child.

[25] Wareham NJ, Rennie KL (1998). The assessment of physical activity in individuals and populations: why try to be more precise about how physical activity is assessed?. Int J Obes.

[26] Higgins JPT, Green S (editors). Cochrane handbook for systematic reviews of interventions, version 5.1.0 (updated March 2011). The Cochrane Collaboration. 2011. Available at http://www.cochrane-handbook.org.

[27] Cohen J. Statistical power analysis in the behavioral sciences. 2nd ed. Hillsdale, NJ: Lawrence Erlbaum Associates, Inc.; 1988.

[28] Deforche B, De Bourdeaudhuij I, D’Hondt E (2009). Objectively measured physical activity, physical activity related personality and body mass index in 6-to 10-year-old children: a cross-sectional study. Int J Behav Nutr Phys Act.

[29] Frömel K, Pelclová J, Skalik K, et al. The association between participation in organised physical activity and level of physical activity and inactivity in adolescent girls. Acta Univ Palacki Olomuc Gymn. 2012;42(1):7–16.

[30] Frömel K, Stelzer J, Groffik D (2008). Physical activity of children ages 6–8: the beginning of school attendance. J Res Child Educ.

[31] Godard C, Roman M, del Pilar Rodriguez M, et al. Variability of physical activity in 4 to 10-year-old children: a study by accelerometry. Arch Argent Pediatr. 2012;110(5):388–93. doi:10.5546/aap.2012.388. 10.5546/aap.2012.eng.388 23070180

[32] Rowlands AV, Pilgrim EL, Eston RG (2008). Patterns of habitual activity across weekdays and weekend days in 9- to 11-year-old children. Prev Med.

[33] Soric M, Misigoj-Durakovic M (2010). Physical activity levels and estimated energy expenditure in overweight and normal-weight 11-year-old children. Acta Paediatr.

[34] Stone MR, Rowlands AV, Eston RG (2009). Characteristics of the activity pattern in normal weight and overweight boys. Prev Med.

[35] Sherar LB, Muhajarine N, Esliger DW (2009). The relationship between girls’ (8–14 years) physical activity and maternal education. Ann Hum Biol.

[36] Basterfield L, Adamson AJ, Pearce MS (2011). Stability of habitual physical activity and sedentary behavior monitoring by accelerometry in 6- to 8-year-olds. J Phys Act Health.

[37] Jurisson A, Jurimae T (1996). The validity of the Godin–Shephard physical activity questionnaire in children. Biol Sport.

[38] Wilkin TJ, Mallam KM, Metcalf BS (2006). Variation in physical activity lies with the child, not his environment: evidence for an ‘activitystat’ in young children (EarlyBird 16). Int J Obes.

[39] Kemp C, Pienaar AE (2011). Physical activity levels and energy expenditure of 9-year-old to 12-year-old overweight and obese children. Health SA Gesondheid.

[40] McManus AM, Chu EY, Yu CC (2011). How children move: activity pattern characteristics in lean and obese Chinese children. J Obes.

[41] Trost SG, Pate RR, Freedson PS (2000). Using objective physical activity measures with youth: how many days of monitoring are needed?. Med Sci Sports Exerc.

[42] Esliger DW, Tremblay MS, Copeland JL, et al. Physical activity profile of Old Order Amish, Mennonite, and contemporary children. Med Sci Sports Exerc. 2010;42(2):296–303. doi:10.1249/MSS.0b013e3181b3afd2. 10.1249/MSS.0b013e3181b3afd2 19927029

[43] Jauregui A, Villalpando S, Rangel-Baltazar E (2011). The physical activity level of Mexican children decreases upon entry to elementary school. Salud Publica Mex.

[44] Silva P, Rute S, Welk G (2011). Seasonal differences in physical activity and sedentary patterns: the relevance of the PA context. J Sports Sci Med.

[45] Rush E, Coppinger T, Obolonkin V (2012). Use of pedometers to identify less active children and time spent in moderate to vigorous physical activity in the school setting. J Sci Med Sport.

[46] Jago R, Fox KR, Page AS (2010). Physical activity and sedentary behaviour typologies of 10–11 year olds. Int J Behav Nutr Phys Act.

[47] Kriemler S, Zahner L, Schindler C (2010). Effect of school based physical activity programme (KISS) on fitness and adiposity in primary schoolchildren: cluster randomised controlled trial. Br Med J.

[48] Nilsson A, Anderssen SA, Andersen LB (2009). Between- and within-day variability in physical activity and inactivity in 9-and 15-year-old European children. Scand J Med Sci Sports.

[49] Panter J, Jones A, Van Sluijs E (2011). The influence of distance to school on the associations between active commuting and physical activity. Ped Exerc Sci.

[50] Taylor RW, Farmer VL, Cameron SL (2011). School playgrounds and physical activity policies as predictors of school and home time activity. Int J Behav Nutr Phys Act.

[51] Fuemmeler BF, Anderson CB, Masse LC (2011). Parent-child relationship of directly measured physical activity. Int J Behav Nutr Phys Act.

[52] Knowles G, Pallan M, Thomas GN (2013). Physical activity and blood pressure in primary school children: a longitudinal study. Hypertension.

[53] Cook NR, Cohen J, Hebert PR (1995). Implications of small reductions in diastolic blood pressure for primary prevention. Arch Intern Med.

[54] Ridgers ND, Saint-Maurice PF, Welk GJ (2011). Differences in physical activity during school recess. J Sch Health.

[55] Erwin H, Abel M, Beighle A (2012). The contribution of recess to children’s school-day physical activity. J Phys Act Health.

[56] Dudley DA, Okely AD, Cotton WG (2012). Physical activity levels and movement skill instruction in secondary school physical education. J Sci Med Sport.

[57] Fairclough S, Stratton G (2005). Physical activity levels in middle and high school physical education: a review. Ped Exerc Sci.

[58] Fairclough SJ, Stratton G (2006). A review of physical activity levels during elementary school physical education. J Teach Phys Educ.

[59] Fairclough SJ, Butcher ZH, Stratton G (2007). Whole-day and segmented-day physical activity variability of northwest England school children. Prev Med.

[60] van Sluijs EM, Fearne VA, Mattocks C (2009). The contribution of active travel to children’s physical activity levels: cross-sectional results from the ALSPAC study. Prev Med.

[61] Doherty AR, Kelly P, Kerr J, et al. Using wearable cameras to categorise type and context of accelerometer-identified episodes of physical activity. Int J Behav Nutr Phys Act. 2013;10. doi:10.1186/1479-5868-10-22. 10.1186/1479-5868-10-22 PMC3615956 23406270

[62] Hodges S, Berry E, Wood K (2011). SenseCam: a wearable camera that stimulates and rehabilitates autobiographical memory. Memory.

[63] Rich C, Griffiths LJ, Dezateux C (2012). Seasonal variation in accelerometer-determined sedentary behaviour and physical activity in children: a review. Int J Behav Nutr Phys Act.

[64] Riddoch CJ, Mattocks C, Deere K (2007). Objective measurement of levels and patterns of physical activity. Arch Dis Child.

[65] Carson V, Spence JC (2010). Seasonal variation in physical activity among children and adolescents: a review. Ped Exerc Sci.

[66] Orme M, Wijndaele K, Sharp SJ (2014). Combined influence of epoch length, cut-point and bout duration on accelerometry-derived physical activity. Int J Behav Nutr Phys Act.

[67] Corder K, van Sluijs EM, Ekelund U (2010). Changes in children’s physical activity over 12 months: longitudinal results from the SPEEDY study. Pediatrics.

[68] O’Connor J, Ball EJ, Steinbeck KS (2003). Measuring physical activity in children: a comparison of four different methods. Ped Exerc Sci.

[69] Corder K, Craggs C, Jones AP, et al. Predictors of change differ for moderate and vigorous intensity physical activity and for weekdays and weekends: a longitudinal analysis. Int J Behav Nutr Phys Act. 2013;10. doi:10.1186/1479-5868-10-69. 10.1186/1479-5868-10-69 PMC3672092 23714688

[70] McMinn AM, Griffin SJ, Jones AP (2013). Family and home influences on children’s after-school and weekend physical activity. Eur J Public Health.

[71] Stanley RM, Ridley K, Dollman J. Correlates of children’s time-specific physical activity: a review of the literature. Int J Behav Nutr Phys Act. 2012;9. doi:10.1186/1479-5868-9-50. 10.1186/1479-5868-9-50 PMC3441809 22546218

[72] Generelo E, Zaragoza J, Julian JA (2011). Physical activity patterns in normal-weight adolescents on week-days and week-ends. J Sports Med Phys Fit.

[73] Aibar A, Bois JE, Generelo E (2013). A cross-cultural study of adolescents’ physical activity levels in France and Spain. Eur J Sport Sci.

[74] De Meester F, De Bourdeaudhuij I, Deforche B (2011). Measuring physical activity using accelerometry in 13- to 15-year-old adolescents: the importance of including non-wear activities. Public Health Nutr.

[75] Cooper AR, Page AS, Foster LJ (2003). Commuting to school: are children who walk more physically active?. Am J Prev Med.

[76] Ford P, Bailey R, Coleman D (2007). Activity levels, dietary energy intake, and body composition in children who walk to school. Ped Exerc Sci.

[77] Gidlow CJ, Cochrane T, Davey R (2008). In-school and out-of-school physical activity in primary and secondary school children. J Sports Sci.

