# Supplementary material for: A Systematic Literature Review with Meta-Analyses of Within- and Between-Day Differences in Objectively Measured Physical Activity in School-Aged Children
Source: Sports Med. 2014 Jul 1;44(10):1427–38. doi: 10.1007/s40279-014-0215-5 (PMC4171592; doi:10.1007/s40279-014-0215-5)
Supplement: Supplementary file 1 — Supplementary material 1 (PDF 217 kb) [file 40279_2014_215_MOESM1_ESM.pdf]

ELECTRONIC SUPPLEMENTARY MATERIAL

A systematic literature review with meta-analyses of within- and between-day differences in objectively measured physical activity in school-aged children

SPORTS MEDICINE

Hannah L Brooke[1], Kirsten Corder[1,*], Andrew J Atkin[1], Esther M F van Sluijs[1]

[1] *UKCRC Centre for Diet and Activity Research (CEDAR) and MRC Epidemiology Unit, University of Cambridge School of Clinical Medicine, Box 285 Institute of Metabolic Science, Cambridge Biomedical Campus, Cambridge, CB2 0QQ, U.K.*

*Corresponding Author

Email: klc29@medschl.cam.ac.uk

Electronic Supplementary Material Table S1. Search strategy conducted in January 2013 of MEDLINE via PubMed, Scopus, Science Citation Index (SCI) via Web of Knowledge, and SPORTDiscus via EBSCOhost limited to papers published post-1990

Theme	Keywords	Mesh terms (PubMed only)
Young people	Children, child, childhood, adolescence, adolescent, teen, teens, teenaged, teenager, teenagers, "young people", youth, youths, boy, boys, girl, girls, "school aged",	"Child", "Adolescent"
Physical activity	"physical activity", "physical activities", exercise, "energy expenditure", sport, sports, "active travel", ("leisure time", AND, activity),	"Motor Activity", "Sports", "Exercise", "Physical Exertion”
Objective measures	Accelerometer, accelerometry, accelerometers, "counts per minute", CPM, triaxial, Actigraph, Yamax, Actiheart, Omron, sensewear, caltrac, walk4life, ideea, actireg, lifecorder, tritrac, genea, stepwatch, actical, actiwatch, rt3, activpal, actimarker, dynaport, CSA, MTI, pedometer, "heart rate", pedometry, pedometers, uniaxial, actigraphy,	"Monitoring, Ambulatory", "Actigraphy"
Time-segments	Hour, hours, hourly, day, days, daily, week, weeks, weekly, morning, afternoon, evening, weekday, weekdays, weekend, school, "break time", "play time", recess, lunchtime	

Themes were combined using AND, keywords were combined using OR. There were no language limitations enforced.

Electronic Supplementary Material Table S2. Study by study descriptive characteristics

Study	Year	Study name	Country	Sample size	Age (years)	Ethnicity	SES	Meta-analysed	Accelerometer	Epoch length (seconds)	MVPA cut-point (cpm[a])	Minimum registered activity for inclusion (minutes)	
												Week days	Weekends
Aibar, et al.[1]	2013	EPAPA	France and Spain	301	14 to 15		Low to medium	Yes	Actigraph, GT3X	15	2296	600	480
Alhassan, et al.[2]	2008	GEMS	USA	293	8 to 10	Majority other than white - African American	Low	Yes	Actigraph, model not stated	60	3000	585	330
Aznar, et al.[3]	2011		Spain	221	9 and 15			Yes	Actigraph, GT1M	15	2000	600	600
Basterfield, et al.[4]	2011	Gateshead Millennium Study	Britain	291	6 to 8			No	Actigraph, GT1M	NR	3200	360	360
Bringolf-Isler, et al.[5]	2009	SCARPOL	Switzerland	169	6 to 14		Medium to high	Yes	CSA/MTI, 7164	60	1950	480	480
Carver, et al.[6]	2008	CLAN	Australia	534	8 to 9 and 13 to 15		Medium to high	Yes	CSA/MTI, 7164	60	Age specific	167	167
Cooper, et al.[7]	2003		Britain	114	9 to 10		Low	Yes	CSA/MTI, 7164	60	Age specific	720	600
De Meester, et al.[8]	2011		Belgium	513	13 to 15		High	Yes	Actigraph, GT1M	60	3200	600	480
Deforche, et al.[9]	2009		Belgium	97	6 to 10			No	CSA/MTI, 7164	60	2000	600	600
Edwardson, et al.[10]	2013		Britain	136	12 to 16	Majority white	Medium to high	Yes	Actigraph, GT1M	5	Age specific	252[b]	540
Esliger, et al.[11]	2010		Canada	413	8 to 13			No	CSA/MTI, 7164	60	Age specific	600	600
Fairclough, et al.[12]	2007		Britain	58	7 to 11			Yes	Actigraph, GT1M	5	1956	600	NA
Fairclough, et al.[13]	2012		Britain	175	10 to 11		Medium to high	Yes	Actigraph, GT1M	5	2000	629	605
Ford, et al.[14]	2007		Britain	239	5 to 11			Yes	CSA/MTI, 7164	60	NA	720	600
Frömel, et al.[15]	2012		Poland	497	16 to 18			No	Actigraph, GT1M	60	1951	600	600
Frömel, et al.[16]	2008		Poland	244	7 to 8			No	Caltrac	NR	NA	NR	NR
Fuemmeler et al.,[17]	2011		USA	45	9 to 11	Majority white	Medium to high	Yes	CSA/MTI, 7164	60	Age specific	600	600
Generelo, et al.[18]	2011		Spain	104	11 to 15			Yes	Actigraph, GT1M	60	Age specific	600	480
Gidlow, et al.[19]	2008	CHAMPS	Britain	446	5 to 16	Majority white	Low	Yes	Actigraph, GT1M	60	Age specific	600	480
Godard, et al.[20]	2012	INTA	Chile	109	4 to 10			No	Actiwatch AW64	NR	900	450	450
Jago, et al.[21]	2010	Bristol 3Ps	Britain	952	10 to 11		Other - IMD score 20.73 ± 16.52	Yes	Actigraph, GT1M	NR	2912	500	500
Jáuregui, et al.[22]	2011		Mexico	217	6 to 7		Low - medium	Both	RT3	60	970	600	600
Jürisson, et al.[23]	1996			24	10 to 13			No	Caltrac	NR	NR	NR	NR
Kemp, et al.[24]	2011		South Africa	24	9 to 12	Majority white		No	Actical	NR	2.7[c]	NR	NR
Kolle, et al.[25]	2010		Norway	1824	9 and 15			Yes	CSA/MTI, 7164	10	2000	480	480
Konharn, et al.[26]	2012		Thailand	186	13 to 18			Yes	Actigraph, GT1M	30	Age specific	600	600
Kriemler, et al.[27]	2010		Switzerland	352	6 to 12		Other - 9% with no formal parental education	Yes	CSA/MTI, 7164	60	2000	720	NA
Lin, et al.,[28]	2012		Taiwan	36	8 to 10			Yes	Actigraph, GT1M	NR	Age specific	NR	NR
Machado-Rodrigues, et al.[29]	2012	MALS	Portuguese	362	13 to 16		Medium to high	Yes	Actigraph, GT1M	60	Age specific	600	600
McLure, et al.[30]	2009		Britain	246	9 to 10		Low to medium	Yes	Actigraph, GT-256	60	1100	180	180
McManus, et al.[31]	2011		Hong Kong	84	7 to 9			No	RT3	1	1860	NR	NR
Miller, et al.[32]	2013	SRTS initiative	USA	193	6 to 12			Yes	Actical	60	NA	NR	NA
Nader, et al.[33]	2008	NICHD SECC	USA	839	9	Majority white	Other - 24% low family income	Yes	CSA, model not stated	60	Age specific	NR	NR
Nilsson, et al.[34]	2009	EYHS	Denmark, Portugal, Estonia, Norway	1954	9 and 15			Yes	CSA/MTI, 7164	60	2000	600	600

Electronic Supplementary Material Table S2. Study by study descriptive characteristics continued

Study	Year	Study name	Country	Sample size	Age (years)	Ethnicity	SES	Meta-analysed	Accelerometer	Epoch length (seconds)	MVPA cut-point (cpm[a])	Minimum registered activity for inclusion (minutes) Week days	Weekends
Owen, et al.[35]	2012	CHASE	Britain	2035	9 to 10	Mixed		Yes	Actigraph, GT1M	5	2000	600	600
Page, et al.[36]	2009	PEACH	Britain	1300	10 to 11		Other - IMD score 27.29 ± 17.84	Yes	Actigraph, GT1M	10	NA	480	480
Panter, et al.[37]	2011	SPEEDY	Britain	1824	9 to 10		Low to medium	Yes	Actigraph, GT1M	5	2000	500	500
Ridgers, et al.[38]	2010	A-CLASS Project	Britain	110	9 to 10			Yes	Actigraph, GT1M	5	Individually calibrated	540	540
Rowlands, et al.[39]	2008		Britain	84	9 to 11			No	Actigraph, GT1M	2	1950	600	480
Rush, et al.[40]	2012		New Zealand	47	8 to 11		Low	No	Actical	15	1500	NR	NA
Sherar, et al.[41]	2009		Canada	77	9 to 14		Medium to high	No	Actical	15	2300	600	600
Silva, et al.[42]	2011		Portuguese	24	10 to 13			No	CSA/MTI, 7164	60	Age specific	480	NA
Sirard, et al.[43]	2008	Team COOL	USA	65	14 to 17	Majority other than white - African American	Low	Yes	Actigraph, GT1M	30	Age specific	600	600
Soric, et al.[44]	2010		Croatia	91	10.5 to 11.5			No	SenseWear Armband	60	3[c]	1260	1260
Stone, et al.[45]	2013	BEAT	Canada	856	10 to 12			Yes	Actigraph, GT1M	NR	4242	600	600
Stone, et al.[46]	2009		Britain	47	8 to 10			No	Actigraph, GT1M	2	3581	600	600
Sulemana, et al.[47]	2006		USA	172	14 to 17	Mixed		Yes	Actigraphy	60	NA	NR	NA
Taylor, et al.[48]	2011	PLAY	New Zealand	427	5 to 13	Majority white	Medium to high	Yes	Actigraph, GT3X	60	1500	480	NA
Treuth, et al.[49]	2007	TAAG	USA	1577	11 to 12	Mixed		Yes	CSA/MTI, 7165	30	1500	360[d]	360[d]
Trost, et al.[50]	2000	Amherst Health and Activity study	USA	381	6 to 12	Majority white		No	CSA/MTI, 7164	60	Age specific	NR	NR
Uvacsek, et al.[51]	2011		Hungary	53	9 to 13		High	Yes	CSA/MTI, 7164	5	2000	671	575
van Sluijs, et al.[52]	2009	ALSPAC	Britain	4688	11	Majority white		Yes	CSA/MTI, 7164	60	3600	600	600
Veitch, et al.[53]	2010	CLASS	Australia	173	8 to 9		Other - varying	Yes	CSA/MTI, 7164	60	Unclear	167	167
Wilkin, et al.[54]	2006		Britain	431	4 to 10			No	CSA/MTI, model not stated	60	1000	NR	NR

[a] If cut-point was reported per epoch length the per minute equivalent was calculated. NR, not reported; NA. not applicable eg. study only measured physical activity on weekdays; SES, socioeconomic status; MVPA, moderate-to-vigorous intensity physical activity; CSA, computer science applications; MTI, manufacturing technologies Inc, [b] valid after-school period; [c] reported as Metabolic equivalents as this was the measure reported by the monitor, [d] at least one day of seven with 360 minutes of data was required

Study	Year	Subgroup	SMD and 95% CI of accelerometer CPM	Weight	SMD and 95% CI of minutes in MVPA	Weight
Aibar, et al.[1]	2012	French Boys	N/A	N/A	0.97 (0.60, 1.35)	1.35
		French Girls	N/A	N/A	0.83 (0.54, 1.12)	1.61
		Spanish Boys	N/A	N/A	0.59 (0.24, 0.94)	1.42
		Spanish Girls	N/A	N/A	0.34 (0.02, 0.67)	1.49
Alhassan, et al.[2]	2008	N/A	-0.35 (-0.51, -0.18)	2.36	N/A	N/A
Aznar, et al.[3]	2011	Younger Boys	N/A	N/A	0.14 (-0.20, 0.48)	1.46
		Younger Girls	N/A	N/A	0.22 (-0.12, 0.55)	1.45
		Older Boys	N/A	N/A	0.68 (0.28, 1.09)	1.24
		Older Girls	N/A	N/A	0.55 (0.08, 1.02)	1.08
Bringolf-Isler, et al.[5]	2009	N/A	0.22 (0.01, 0.44)	2.11	N/A	N/A
Cooper, et al.[7]	2003	Boys Walk	0.50 (0.04, 0.97)	1.08	0.21 (-0.24, 0.67)	1.11
		Boys Car	0.02 (-0.57, 0.61)	0.78	-0.15 (-0.74, 0.44)	0.83
		Girls Walk	0.18 (-0.27, 0.64)	1.10	0.27 (-0.19, 0.73)	1.11
		Girls Car	0.32 (-0.34, 0.97)	0.67	0.27 (-0.38, 0.93)	0.72
De Meester, et al.[8]	2011	Boys	N/A	N/A	0.36 (0.18, 0.54)	1.98
		Girls	N/A	N/A	0.55 (0.38, 0.73)	1.98
Fairclough, et al.[13]	2012	Boys	0.38 (0.07, 0.70)	1.61	N/A	N/A
		Girls	0.35 (0.07, 0.63)	1.76	N/A	N/A
Ford, et al.[14]	2007	Walk Boys	-0.25 (-0.62, 0.11)	1.42	N/A	N/A
		Walk Girls	-0.19 (-0.59, 0.21)	1.30	N/A	N/A
		Car Boys	0.00 (-0.34, 0.34)	1.51	N/A	N/A
		Car Girls	0.28 (-0.06, 0.63)	1.49	N/A	N/A
Fuemmeler, et al.[17]	2011	Boys	0.22 (-0.36, 0.80)	0.80	0.42 (-0.16, 1.01)	0.84
		Girls	-0.42 (-1.01, 0.18)	0.77	-0.18 (-0.78, 0.41)	0.83
Generelo, et al.[18]	2011	Boys	N/A	N/A	0.57 (0.21, 0.92)	1.40
		Girls	N/A	N/A	0.48 (0.04, 0.93)	1.14
Gidlow, et al.[19]	2008	Primary school	-0.13 (-0.31, 0.06)	2.27	N/A	N/A
		Secondary school	0.26 (0.07, 0.45)	2.22	N/A	N/A
Jáuregui, et al.[22]	2011	N/A	N/A	N/A	0.05 (-0.14, 0.24)	1.94
Kolle, et al.[25]	2010	Younger Boys	0.24 (0.13, 0.35)	2.58	N/A	N/A
		Younger Girls	0.15 (0.03, 0.28)	2.55	N/A	N/A
		Older Boys	0.46 (0.31, 0.61)	2.41	N/A	N/A
		Older Girls	0.23 (0.08, 0.38)	2.44	N/A	N/A
Konharn, et al.[26]	2012	Boys	N/A	N/A	0.45 (0.15, 0.74)	1.60
		Girls	N/A	N/A	0.56 (0.27, 0.85)	1.60
Lin, et al.[28]	2012	Overweight	N/A	N/A	0.60 (-0.06, 1.27)	0.70
		Normal Weight	N/A	N/A	0.17 (-0.49, 0.82)	0.72
Machado-Rodrigues, et al.[29]	2012	Younger Boys	N/A	N/A	1.12 (0.82, 1.42)	1.58
		Older Boys	N/A	N/A	0.77 (0.42, 1.13)	1.39
		Younger Girls	N/A	N/A	1.05 (0.78, 1.33)	1.65
		Older Girls	N/A	N/A	1.05 (0.72, 1.38)	1.49
McLure, et al.[30]	2009	Boys	0.13 (-0.14, 0.40)	1.82	0.49 (0.22, 0.77)	1.66
		Girls	0.01 (-0.23, 0.24)	2.01	0.59 (0.35, 0.83)	1.78
Nader, et al.[33]	2008	Boys	N/A	N/A	0.11 (-0.03, 0.24)	2.09
		Girls	N/A	N/A	0.00 (-0.13, 0.13)	2.10
Nilsson, et al.[34]	2009	Denmark Younger Boys	0.33 (0.09, 0.56)	2.00	0.53 (0.30, 0.77)	1.78
		Denmark Younger Girls	0.00 (-0.22, 0.22)	2.08	0.18 (-0.04, 0.40)	1.84
		Portugal Younger Boys	-0.13 (-0.35, 0.10)	2.05	0.11 (-0.11, 0.34)	1.82
		Portugal Younger Girls	-0.29 (-0.52, -0.06)	2.00	0.03 (-0.21, 0.26)	1.79
		Estonia Younger Boys	-0.04 (-0.27, 0.19)	2.05	0.17 (-0.05, 0.40)	1.82
		Estonia Younger Girls	-0.07 (-0.29, 0.16)	2.04	0.26 (0.03, 0.49)	1.81
		Norway Younger Boys	0.27 (0.04, 0.49)	2.05	0.73 (0.50, 0.97)	1.80
		Norway Younger Girls	0.21 (-0.02, 0.45)	2.00	0.64 (0.40, 0.88)	1.77
		Denmark Older Boys	0.16 (-0.14, 0.46)	1.69	0.21 (-0.09, 0.51)	1.57
		Denmark Older Girls	0.24 (-0.02, 0.50)	1.86	0.36 (0.10, 0.63)	1.69
		Portugal Older Boys	-0.06 (-0.37, 0.25)	1.63	0.24 (-0.07, 0.55)	1.53
		Portugal Older Girls	-0.03 (-0.34, 0.27)	1.67	0.53 (0.22, 0.84)	1.54
		Estonia Older Boys	0.20 (-0.08, 0.47)	1.79	0.51 (0.22, 0.79)	1.63
		Estonia Older Girls	0.14 (-0.07, 0.35)	2.12	0.52 (0.31, 0.74)	1.86
		Norway Older Boys	0.46 (0.09, 0.83)	1.40	0.84 (0.46, 1.22)	1.32
		Norway Older Girls	0.42 (0.11, 0.73)	1.63	0.88 (0.56, 1.21)	1.49
Owen, et al.[35]	2012	Walking/Cycling	0.27 (0.20, 0.35)	2.72	0.29 (0.21, 0.36)	2.23
		Public Transport	0.33 (0.11, 0.55)	2.08	0.50 (0.28, 0.72)	1.83
		Car	0.26 (0.13, 0.38)	2.52	0.35 (0.22, 0.48)	2.12
Page, et al.[36]	2009	Boys	0.04 (-0.08, 0.17)	2.54	N/A	N/A
		Girls	-0.16 (-0.28, -0.04)	2.55	N/A	N/A
Panter, et al.[37]	2011	Boys	-0.11 (-0.21, -0.02)	2.64	-0.03 (-0.13, 0.07)	2.18
		Girls	-0.17 (-0.25, -0.08)	2.68	-0.05 (-0.14, 0.04)	2.20
Ridgers, et al.[38]	2010	Boys	N/A	N/A	-0.10 (-0.52, 0.31)	1.23
		Girls	N/A	N/A	0.24 (-0.11, 0.58)	1.43
Sirard, et al.[43]	2008	N/A	0.12 (-0.22, 0.47)	1.50	N/A	N/A
Stone, et al.[46]	2013	<9Hrs Sleep	0.54 (0.31, 0.76)	2.06	0.71 (0.49, 0.94)	1.82
		9-10Hrs Sleep	0.35 (0.18, 0.52)	2.32	0.46 (0.29, 0.63)	1.99
		>10Hrs Sleep	0.51 (0.37, 0.65)	2.46	0.77 (0.63, 0.91)	2.07
Treuth, et al.[49]	2007	Normal Weight	N/A	N/A	0.19 (0.10, 0.28)	2.21
		At-Risk And Overweight	N/A	N/A	0.23 (0.11, 0.35)	2.13
Uvacsek, et al.[51]	2011	N/A	0.86 (0.47, 1.26)	1.29	N/A	N/A
		Boys	N/A	N/A	1.07 (0.51, 1.63)	0.88
		Girls	N/A	N/A	1.52 (0.89, 2.15)	0.76
van Sluijs, et al.[52]	2009	Boys	0.14 (0.08, 0.20)	2.76	0.32 (0.26, 0.38)	2.25
		Girls	0.02 (-0.04, 0.08)		0.30 (0.24, 0.35)	2.25
Pooled SMD			0.14 (0.08, 0.20)	100.0	0.42 (0.35, 0.49)	100.0
I-squared			83.5%, p < 0.001		86.1%, p < 0.001	

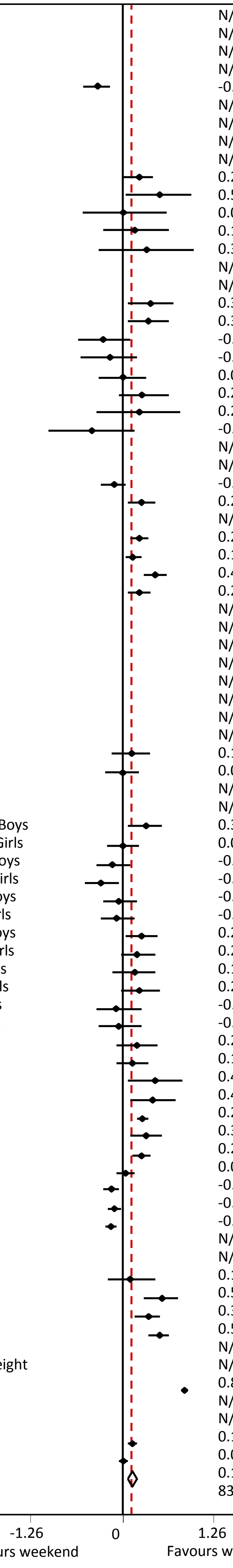

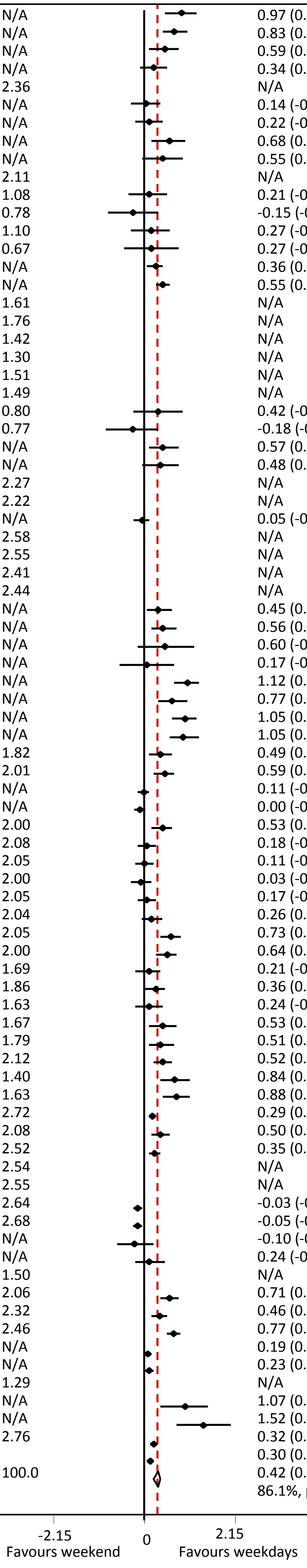

Electronic Supplementary Material Figure S1. Forest plots showing standardised mean differences (SMD) and 95% confidence intervals (CI) between week day and weekend day physical activity measured by accelerometer in average counts per minute (CPM) and minutes in moderate-to-vigorous intensity physical activity (MVPA). SMD is reported in units of standard deviation; 0.2, 0.5 and 0.8 respectively represent small, moderate and large effects. I^2 is a measure of heterogeneity between studies.

Study	Year	Subgroup	SMD and 95%CI of accelerometer CPM	Weight	SMD and 95%CI of minutes in MVPA	Weight
Fairclough, et al.[12]	2007	Boys	N/A	N/A	2.51 (1.84, 3.18)	2.68
		Girls	N/A	N/A	2.04 (1.38, 2.70)	2.69
Ford, et al.[14]	2007	Walk Boys	0.17 (-0.19, 0.53)	2.61	N/A	N/A
		Walk Girls	0.46 (0.06, 0.86)	2.53	N/A	N/A
		Car Boys	0.68 (0.33, 1.04)	2.63	N/A	N/A
		Car Girls	0.13 (-0.22, 0.47)	2.64	N/A	N/A
Gidlow, et al.[19]	2008	Primary school	-0.35 (-0.53, -0.16)	2.90	N/A	N/A
		Secondary school	-1.45 (-1.66, -1.24)	2.86	N/A	N/A
Jago, et al.[21]	2010	Hi Act - Low Sed	-0.79 (-0.95, -0.64)	2.94	0.67 (0.52, 0.82)	3.68
		Low Act - Med Sed	-0.68 (-0.82, -0.54)	2.95	0.76 (0.62, 0.90)	3.69
		Hi Act - Hi Sed	-0.57 (-0.79, -0.34)	2.84	0.78 (0.55, 1.01)	3.59
Kriemler, et al.[27]	2010	Intervention Group	0.21 (0.02, 0.40)	2.89	-1.31 (-1.51, -1.10)	3.62
		Control Group	0.20 (-0.04, 0.44)	2.82	-1.58 (-1.85, -1.30)	3.52
Lin, et al.[28]	2012	Overweight	N/A	N/A	1.05 (0.35, 1.74)	2.61
		Normal Weight	N/A	N/A	0.70 (0.03, 1.38)	2.67
Miller, et al.[32]	2013	Boys	-0.34 (-0.64, -0.04)	2.72	N/A	N/A
		Girls	-0.12 (-0.39, 0.15)	2.78	N/A	N/A
Nilsson, et al.[34]	2009	Denmark Younger Boys	0.74 (0.50, 0.98)	2.82	0.64 (0.40, 0.88)	3.57
		Denmark Younger Girls	0.21 (-0.01, 0.43)	2.85	0.27 (0.05, 0.49)	3.60
		Portugal Younger Boys	-0.35 (-0.58, -0.13)	2.84	-0.58 (-0.81, -0.35)	3.58
		Portugal Younger Girls	-0.36 (-0.59, -0.12)	2.83	-0.58 (-0.82, -0.34)	3.57
		Estonia Younger Boys	-0.08 (-0.30, 0.15)	2.84	-0.19 (-0.42, 0.04)	3.59
		Estonia Younger Girls	-0.06 (-0.29, 0.16)	2.84	-0.10 (-0.32, 0.13)	3.59
		Norway Younger Boys	0.19 (-0.04, 0.41)	2.84	0.22 (-0.01, 0.44)	3.59
		Norway Younger Girls	-0.05 (-0.28, 0.18)	2.83	0.24 (0.01, 0.48)	3.58
		Denmark Older Boys	0.21 (-0.09, 0.51)	2.72	0.48 (0.18, 0.79)	3.47
		Denmark Older Girls	-0.22 (-0.48, 0.04)	2.79	0.07 (-0.20, 0.33)	3.54
		Portugal Older Boys	-0.10 (-0.41, 0.22)	2.70	0.08 (-0.23, 0.40)	3.45
		Portugal Older Girls	-0.02 (-0.33, 0.28)	2.71	0.34 (0.04, 0.65)	3.46
		Estonia Older Boys	-0.20 (-0.48, 0.08)	2.76	-0.07 (-0.35, 0.21)	3.51
		Estonia Older Girls	-0.28 (-0.49, -0.07)	2.86	0.00 (-0.21, 0.21)	3.61
		Norway Older Boys	-0.22 (-0.59, 0.14)	2.60	-0.04 (-0.40, 0.33)	3.36
		Norway Older Girls	-0.38 (-0.69, -0.07)	2.70	0.11 (-0.20, 0.42)	3.46
Panter, et al.[37]	2011	Boys	-0.65 (-0.75, -0.55)	2.98	0.12 (0.03, 0.22)	3.72
		Girls	-0.97 (-1.06, -0.88)	2.99	-0.21 (-0.29, -0.12)	3.73
Sulemana, et al.[47]	2006	Normal	-0.89 (-1.21, -0.57)	2.69	N/A	N/A
		At-risk-of-overweight	-0.77 (-1.22, -0.33)	2.44	N/A	N/A
		Overweight	-0.90 (-1.33, -0.47)	2.47	N/A	N/A
Taylor, et al.[48]	2011	Boys	-0.32 (-0.51, -0.14)	2.90	0.10 (-0.09, 0.28)	3.65
		Girls	-0.46 (-0.66, -0.26)	2.88	-0.12 (-0.32, 0.07)	3.63
Pooled SMD			-0.24 (-0.39, -0.08)	100.00	0.17 (-0.03, 0.38)	100.00
I-squared			94.8%, p < 0.001		96.3% p < 0.001	

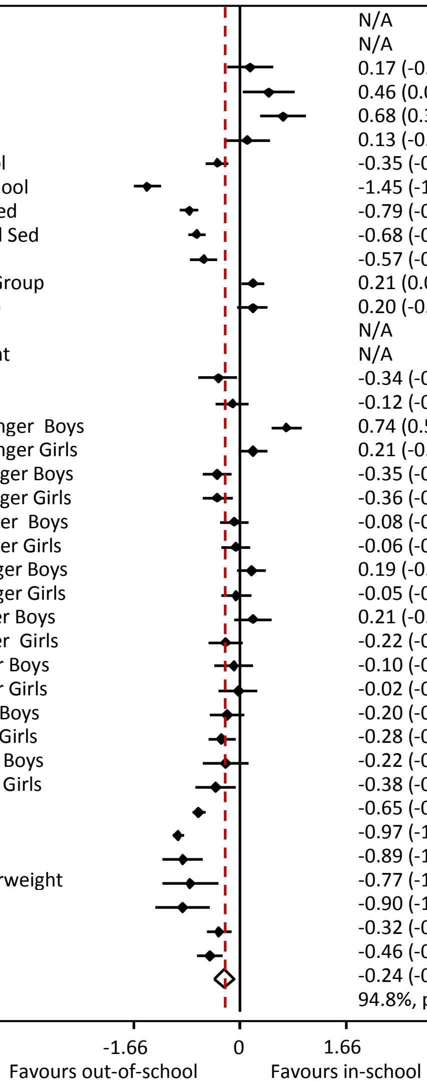

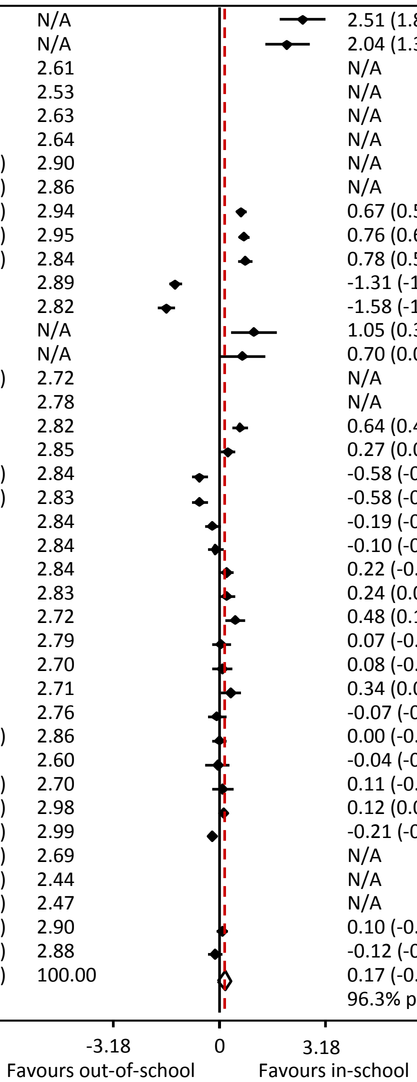

Electronic Supplementary Material Figure S2. Forest plots showing standardised mean differences (SMD) and 95% confidence intervals (CI) between in-school and out-of-school physical activity measured by accelerometer in average counts per minute (CPM) and minutes in moderate-to-vigorous intensity physical activity (MVPA). SMD is reported in units of standard deviation; 0.2, 0.5 and 0.8 respectively represent small, moderate and large effects. I^2 is a measure of heterogeneity between studies.

Study	Year	Subgroup	SMD and 95% CI of accelerometer CPM	Weight	SMD and 95% CI of minutes in MVPA	Weight
Carver, et al.[6]	2008	Younger Boys	N/A	N/A	1.19 (0.86, 1.52)	3.48
		Younger Girls	N/A	N/A	1.10 (0.81, 1.39)	3.55
		Older Boys	N/A	N/A	0.64 (0.43, 0.85)	3.68
		Older Girls	N/A	N/A	0.41 (0.19, 0.63)	3.67
Edwardson, et al.[10]	2012	Boys	N/A	N/A	1.19 (0.81, 1.56)	3.39
		Girls	N/A	N/A	0.67 (0.34, 1.01)	3.46
Ford, et al.[14]	2007	Walk Boys	0.50 (0.13, 0.86)	2.99	N/A	N/A
		Walk Girls	0.51 (0.11, 0.91)	2.7	N/A	N/A
		Car Boys	0.35 (0.01, 0.70)	3.18	N/A	N/A
		Car Girls	-0.03 (-0.38, 0.31)	3.18	N/A	N/A
Fuemmeler, et al.[17]	2011	Boys	-0.68 (-1.27, -0.08)	1.63	2.05 (1.34, 2.77)	2.58
		Girls	-0.47 (-1.07, 0.13)	1.62	2.10 (1.36, 2.84)	2.52
Gidlow, et al.[19]	2008	Primary school	-0.02 (-0.20, 0.17)	4.83	N/A	N/A
		Secondary school	-0.73 (-0.92, -0.53)	4.67	N/A	N/A
Lin, et al.[28]	2012	Overweight	N/A	N/A	1.01 (0.31, 1.70)	2.63
		Normal Weight	N/A	N/A	1.34 (0.62, 2.07)	2.56
Nilsson, et al.[34]	2009	Denmark Younger Boys	-0.01 (-0.25, 0.22)	4.27	0.90 (0.66, 1.15)	3.63
		Denmark Younger Girls	0.07 (-0.15, 0.29)	4.42	0.88 (0.65, 1.11)	3.65
		Portugal Younger Boys	-0.08 (-0.30, 0.15)	4.35	0.97 (0.74, 1.21)	3.64
		Portugal Younger Girls	0.09 (-0.14, 0.33)	4.27	0.92 (0.67, 1.16)	3.63
		Estonia Younger Boys	-0.04 (-0.26, 0.19)	4.35	0.83 (0.59, 1.06)	3.64
		Estonia Younger Girls	0.00 (-0.23, 0.22)	4.33	0.89 (0.65, 1.13)	3.64
		Norway Younger Boys	-0.17 (-0.39, 0.06)	4.36	0.85 (0.62, 1.09)	3.64
		Norway Younger Girls	-0.24 (-0.48, -0.01)	4.25	1.06 (0.81, 1.31)	3.62
		Denmark Older Boys	-0.09 (-0.39, 0.21)	3.59	0.77 (0.46, 1.08)	3.52
		Denmark Older Girls	-0.35 (-0.61, -0.08)	3.95	0.65 (0.38, 0.92)	3.59
		Portugal Older Boys	0.01 (-0.30, 0.32)	3.47	0.98 (0.65, 1.31)	3.48
		Portugal Older Girls	0.03 (-0.28, 0.33)	3.54	0.82 (0.51, 1.14)	3.5
		Estonia Older Boys	-0.27 (-0.55, 0.01)	3.8	0.60 (0.32, 0.89)	3.56
		Estonia Older Girls	-0.23 (-0.44, -0.02)	4.5	0.57 (0.36, 0.79)	3.67
		Norway Older Boys	-0.41 (-0.77, -0.04)	2.98	0.55 (0.18, 0.92)	3.39
		Norway Older Girls	-0.42 (-0.73, -0.10)	3.46	0.67 (0.35, 0.98)	3.5
Panter, et al.[37]	2011	Boys	-0.06 (-0.16, 0.04)	5.63	1.81 (1.69, 1.92)	3.79
		Girls	-0.09 (-0.18, -0.01)	5.71	1.80 (1.69, 1.90)	3.8
Veitch, et al.[53]	2010	N/A	N/A	N/A	2.05 (1.79, 2.31)	3.6
Pooled SMD			-0.10 (-0.19, -0.01)	100	1.02 (0.82, 1.23)	100
I-squared			73.3%, p < 0.001		94.6%, p < 0.001	

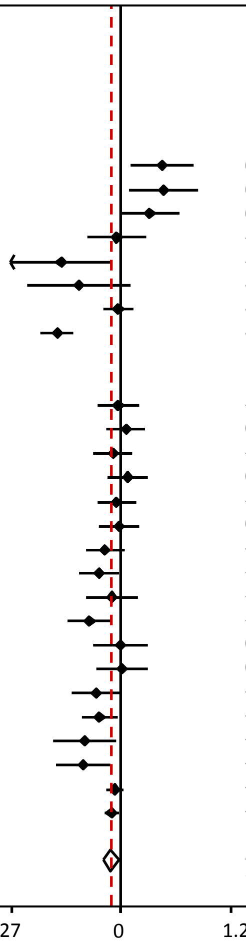

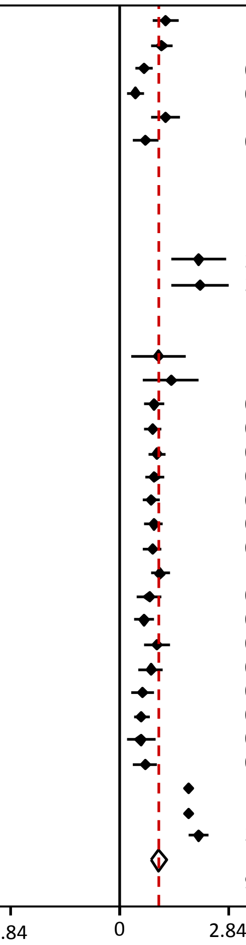

Electronic Supplementary Material Figure S3. Forest plots showing standardised mean differences (SMD) and 95% confidence intervals (CI) between out-of-school and weekend physical activity measured by accelerometer in average counts per minute (CPM) and minutes in moderate-to-vigorous intensity physical activity (MVPA). SMD is reported in units of standard deviation; 0.2, 0.5 and 0.8 respectively represent small, moderate and large effects. I^2 is a measure of heterogeneity between studies.

Electronic Supplementary Material Table S3a. Summary of results of meta-regression analyses in univariate models

		Weekday vs weekend day					In-school vs out-of-school					Weekend vs out-of-school				
		B Coef.	95% CI		P - value	Adjusted R-squared (%)	B Coef.	95% CI		P - value	Adjusted R-squared (%)	B Coef.	95% CI		P - value	Adjusted R-squared (%)
TPA	**Age**	0.03	-0.001	0.05	0.061	7.46	-0.05	-0.11	-0.003	**0.04**	8.83	-0.05	-0.08	-0.02	**0.003**	46.81
	**Gender**															
	Both	Ref					Ref					Ref				
	Boy	-0.17	-0.33	-0.009	**0.039**	25.24	0.46	0.04	0.89	**0.033**	9.31	0.31	-0.09	0.70	0.124	7.57
	Girl	-0.28	-0.44	-0.12	**0.001**		0.20	-0.21	0.61	0.329		0.28	-0.12	0.67	0.161	
	**Global region**															
	Europe	Ref					Ref					Ref				
	North America	0.06	-0.15	0.27	0.558	-0.58	-0.42	-0.89	0.05	0.077	3.16	-0.49	-1.06	0.08	0.089	6.19
	Other	omitted					-0.22	-0.90	0.45	0.506		omitted				
MVPA	**Age**	0.06	0.03	0.09	**<0.001**	30.1	-0.02	-0.13	0.09	0.738	-4.26	-0.09	-0.15	-0.04	**0.002**	33.05
	**Gender**															
	Both	Ref					Ref					Ref				
	Boy	-0.02	-0.28	0.24	0.871	-4.75	0.48	-0.37	1.33	0.258	-3.46	-1.04	-1.91	-0.17	**0.021**	20.10
	Girl	-0.02	-0.28	0.24	0.876		0.29	-0.58	1.16	0.496		-1.12	-1.99	-0.25	**0.014**	
	**Global region**															
	Europe	Ref					Ref					Ref				
	North America	-0.14	-0.36	0.09	0.235	-1.52	0.23	-0.67	1.12	0.609	-3.13	1.15	0.34	1.95	**0.007**	14.35
	Other	-0.11	-0.43	0.22	0.523		omitted					0.16	-0.23	0.55	0.408	

TPA, total physical activity; MVPA, moderate-to-vigorous intensity physical activity; B Coef, Beta Coefficient; 95% CI, 95% Confidence interval; Ref, reference category. Bold p - values indicate a significant association of the potential effect modifier (age, gender, and global region of study setting) with the standardised mean difference in physical activity between time-segments. Significantly factors were combined in a multivariate model where appropriate (**Electronic Supplementary Material Table S3b**). Adjusted R-squared indicates the proportion of between-study variance explained by the covariates. Negative adjusted R-squared indicates that the covariates explain less of the heterogeneity than would be expected by chance. In several models global region 'Other' was omitted due to co-linearity.

Electronic Supplementary Material Table S3b. Summary of results of meta-regression analyses in multivariate models

		B Coef.	95% CI		P - value	Adjusted R-squared (%)	P-value for joint test of all covariates
In-school vs out-of-school TPA	**Age**	-0.06	-0.11	-0.01	0.023	20.33	0.0175
	**Gender**						
	Both						
	Boy	0.51	0.11	0.91	0.015		
	Girl	0.29	-0.10	0.68	0.142		
Weekend vs out-of-school VMPA	**Age**	-0.07	-0.12	-0.02	0.005	53.05	0.0006
	**Gender**						
	Both	Ref					
	Boy	-0.97	-1.70	-0.23	0.012		
	Girl	-1.04	-1.78	-0.29	0.008		
	**Global region**						
	Europe	Ref					
	North America	1.01	0.30	1.72	0.007		
	Other	-0.13	-0.46	0.21	0.437		

TPA, total physical activity; MVPA, moderate-to-vigorous intensity physical activity; B Coef, Beta Coefficient; 95% CI, 95% Confidence interval; Ref, reference category. Adjusted R-squared indicates the proportion of between-study variance explained by the covariates.

Electronic Supplementary Material Table S4. Summary of sensitivity analyses assuming differing degrees of correlation between time-segments

		**Correlation r=0.0**			**Correlation r=0.2**			**Correlation r=0.5**			**Correlation r=0.8**		
**Outcome**	**Time-segment comparison**	**Pooled SMD**	**95% CI of pooled SMD**		**Pooled SMD**	**95% CI of pooled SMD**		**Pooled SMD**	**95% CI of pooled SMD**		**Pooled SMD**	**95% CI of pooled SMD**	
			Lower	Upper		Lower	Upper		Lower	Upper		Lower	Upper
TPA	Weekdays	0.14	0.08	0.20	0.14	0.08	0.20	0.14	0.08	0.20	0.14	0.09	0.20
	Weekend		Ref			Ref			Ref			Ref	
MVPA	Weekdays	0.42	0.35	0.49	0.41	0.35	0.48	0.42	0.36	0.48	0.43	0.37	0.49
	Weekend		Ref			Ref			Ref			Ref	
TPA	In-school	-0.24	-0.40	-0.08	-0.24	-0.39	-0.08	-0.23	-0.39	-0.08	-0.23	-0.38	-0.08
	Out-of-school		Ref			Ref			Ref			Ref	
MVPA	In-school	0.17	-0.03	0.38	0.14	-0.04	0.33	0.17	-0.02	0.35	0.19	0.02	0.37
	Out-of-school		Ref			Ref			Ref			Ref	
TPA	Weekend	-0.10	-0.19	-0.01	-0.10	-0.19	-0.02	-0.10	-0.18	-0.02	-0.10	-0.18	-0.02
	Out-of-school		Ref			Ref			Ref			Ref	
MVPA	Weekend	1.02	0.82	1.23	1.01	0.82	1.20	1.02	0.83	1.21	1.03	0.84	1.22
	Out-of-school		Ref			Ref			Ref			Ref	

TPA, Total physical activity; MVPA, moderate-to-vigorous intensity physical activity; SMD, Standardised mean difference; 95% CI, 95% Confidence interval; Ref, Reference time-segment. SMD is reported in units of standard deviation.

Electronic Supplementary Material References

1. Aibar A, Bois JE, Generelo E, Zaragoza Casterad J, Paillard T. A cross-cultural study of adolescents' physical activity levels in France and Spain. Eur J Sport Sci. 2013;13(5):551-8.
2. Alhassan S, Sirard JR, Spencer TR, Varady A, Robinson TN. Estimating Physical Activity From Incomplete Accelerometer Data in Field Studies. J Phys Act Health. 2008;5(Suppl 1):S112-25.
3. Aznar S, Naylor PJ, Silva P, Perez M, Angulo T, Laguna M et al. Patterns of physical activity in Spanish children: a descriptive pilot study. Child Care Health Dev. 2011;37(3):322-8.
4. Basterfield L, Adamson AJ, Pearce MS, Reilly JJ. Stability of Habitual Physical Activity and Sedentary Behavior Monitoring by Accelerometry in 6-to 8-Year-Olds. J Phys Act Health. 2011;8(4):543-7.
5. Bringolf-Isler B, Grize L, Maeder U, Ruch N, Sennhauser FH, Braun-Fahrlaender C. Assessment of intensity, prevalence and duration of everyday activities in Swiss school children: a cross-sectional analysis of accelerometer and diary data. Int J Behav Nutr Phys Act. 2009;6:50. doi:10.1186/1479-5868-6-50.
6. Carver A, Timperio AF, Crawford DA. Neighborhood road environments and physical activity among youth: The CLAN study. J Urban Health. 2008;85(4):532-44. doi:10.1007/s11524-008-9284-9.
7. Cooper AR, Page AS, Foster LJ, Qahwaji D. Commuting to school - Are children who walk more physically active? Am J Prev Med. 2003;25(4):273-6. doi:10.1016/s0749-3797(03)00205-8.
8. De Meester F, De Bourdeaudhuij I, Deforche B, Ottevaere C, Cardon G. Measuring physical activity using accelerometry in 13-15-year-old adolescents: the importance of including non-wear activities. Public Health Nutr. 2011;14(12):2124-33. doi:10.1017/s1368980011001868.
9. Deforche B, De Bourdeaudhuij I, D'Hondt E, Cardon G. Objectively measured physical activity, physical activity related personality and body mass index in 6-to 10-yr-old children: a cross-sectional study. Int J Behav Nutr Phys Act. 2009;6:25. doi:10.1186/1479-5868-6-25.
10. Edwardson CL, Gorely T, Pearson N, Atkin A. Sources of activity-related social support and adolescents' objectively measured after-school and weekend physical activity: gender and age differences. J Phys Act Health. 2013;10(8):1153-8.
11. Esliger DW, Tremblay MS, Copeland JL, Barnes JD, Huntington GE, Bassett DR, Jr. Physical Activity Profile of Old Order Amish, Mennonite, and Contemporary Children. Med Sci Sports Exerc. 2010;42(2):296-303. doi:10.1249/MSS.0b013e3181b3afd2.
12. Fairclough SJ, Butcher ZH, Stratton G. Whole-day and segmented-day physical activity variability of northwest England school children. Prev Med. 2007;44(5):421-5. doi:10.1016/j.ypmed.2007.01.002.
13. Fairclough SJ, Ridgers ND, Welk G. Correlates of Children's Moderate and Vigorous Physical Activity During Weekdays and Weekends. J Phys Act Health. 2012;9(1):129-37.
14. Ford P, Bailey R, Coleman D, Woolf-May K, Swaine I. Activity levels, dietary energy intake, and body composition in children who walk to school. Pediatr Exerc Sci. 2007;19(4):393-407.
15. Frömel K, Pelclová J, Skalik K, Nováková Lokvencová P, Mitáš J. The association between participation in organised physical activity and level of physical activity and inactivity in adolescent girls. Acta Universitatis Palackianae Olomucensis Gymnica. 2012;42(1):7-16.
16. Frömel K, Stelzer J, Groffik D, Ernest J. Physical activity of children ages 6-8: The beginning of school attendance. J Res Child Educ. 2008;23(1):29-40.

17. Fuemmeler BF, Anderson CB, Masse LC. Parent-child relationship of directly measured physical activity. Int J Behav Nutr Phys Act. 2011;8:17. doi:10.1186/1479-5868-8-17.
18. Generelo E, Zaragoza J, Julian JA, Abarca-Sos A, Murillo B. Physical activity patterns in normal-weight adolescents on week-days and week-ends. J Sports Med Phys Fitness. 2011;51(4)647-653.
19. Gidlow CJ, Cochrane T, Davey R, Smith H. In-school and out-of-school physical activity in primary and secondary school children. J Sports Sci. 2008;26(13):1411-9. doi:10.1080/02640410802277445.
20. Godard C, Roman M, del Pilar Rodriguez M, Leyton B, Salazar G. Variability of physical activity in 4 to 10-year-old children: a study by accelerometry. Arch Argent Pediatr. 2012;110(5):388-93. doi:10.5546/aap.2012.388.
21. Jago R, Fox KR, Page AS, Brockman R, Thompson JL. Physical activity and sedentary behaviour typologies of 10-11 year olds. Int J Behav Nutr Phys Act. 2010;7:59. doi:1479-5868-7-59
22. Jauregui A, Villalpando S, Rangel-Baltazar E, Castro-Hernandez J, Lara-Zamudio Y, Mendez-Gomez-Humaran I. The physical activity level of Mexican children decreases upon entry to elementary school. Salud Publica Mex. 2011;53(3):228-236.
23. Jurisson A, Jurimae T. The validity of the Godin-Shephard physical activity questionnaire in children. Biol Sport. 1996;13(4):291-5.
24. Kemp C, Pienaar AE. Physical activity levels and energy expenditure of 9-year-old - 12-year-old overweight and obese children. Health SA Gesondheid. 2011;16(1):557. doi:10.4102/hsag.v16i1.557
25. Kolle E, Steene-Johannessen J, Andersen LB, Anderssen SA. Objectively assessed physical activity and aerobic fitness in a population-based sample of Norwegian 9- and 15-year-olds. Scand J Med Sci Sports. 2010;20(1):e41-7. doi:10.1111/j.1600-0838.2009.00892.x.
26. Konharn K, Santos MP, Ribeiro JC. Differences Between Weekday and Weekend Levels of Moderate-to-Vigorous Physical Activity in Thai Adolescents. Asia Pac J Public Health. 2012. doi:10.1177/1010539512459946.
27. Kriemler S, Zahner L, Schindler C, Meyer U, Hartmann T, Hebestreit H et al. Effect of school based physical activity programme (KISS) on fitness and adiposity in primary schoolchildren: cluster randomised controlled trial. BMJ. 2010;340:c785. doi:10.1136/bmj.c785.
28. Lin C-Y, Su C-T, Ma H-I. Physical Activity Patterns and Quality of Life of Overweight Boys: A Preliminary Study. Hong Kong J Occupational Therapy. 2012;22(1):31-7. doi:10.1016/j.hkjot.2012.06.001.
29. Machado-Rodrigues AM, Coelho ESMJ, Mota J, Padez C, Martins RA, Cumming SP et al. Urban-rural contrasts in fitness, physical activity, and sedentary behaviour in adolescents. Health Promot Int. 2012. doi:10.1093/heapro/das054.
30. McLure SA, Summerbell CD, Reilly JJ. Objectively measured habitual physical activity in a highly obesogenic environment. Child Care Health Dev. 2009;35(3):369-75. doi:10.1111/j.1365-2214.2009.00946.x.
31. McManus AM, Chu EYW, Yu CCW, Hu Y. How children move: activity pattern characteristics in lean and obese chinese children. J Obes. 2011;2011:679328. doi:10.1155/2011/679328.
32. Miller WC, Redmond JG, Vaux-Bjerke AT. Activity Patterns and Perceptions About Active Transport to School. Am J Health Behav. 2013;37(2):190-8. doi:10.5993/ajhb.37.2.6.
33. Nader PR, Bradley RH, Houts RM, McRitchie SL, O'Brien M. Moderate-to-vigorous physical activity from ages 9 to 15 years. JAMA. 2008;300(3):295-305. doi:300/3/295

34. Nilsson A, Anderssen SA, Andersen LB, Froberg K, Riddoch C, Sardinha LB et al. Between- and within-day variability in physical activity and inactivity in 9-and 15-year-old European children. Scand J Med Sci Sports. 2009;19(1):10-8. doi:10.1111/j.1600-0838.2007.00762.x.
35. Owen CG, Nightingale CM, Rudnicka AR, van Sluijs EMF, Ekelund U, Cook DG et al. Travel to School and Physical Activity Levels in 9-10 Year-Old UK Children of Different Ethnic Origin; Child Heart and Health Study in England (CHASE). Plos One. 2012;7(2)e30932. doi:10.1371/journal.pone.0030932.
36. Page AS, Cooper AR, Griew P, Davis L, Hillsdon M. Independent mobility in relation to weekday and weekend physical activity in children aged 10-11 years: The PEACH Project. Int J Behav Nutr Phys Act. 2009;6:2. doi:10.1186/1479-5868-6-2.
37. Panter J, Jones A, Van Sluijs E, Griffin S. The Influence of Distance to School on the Associations Between Active Commuting and Physical Activity. Pediatr Exerc Sci. 2011;23(1):72-86.
38. Ridgers ND, Graves LEF, Foweather L, Stratton G. Examining Influences on Boy's and Girls' Physical Activity Patterns: The A-CLASS Project. Pediatr Exerc Sci. 2010;22(4):638-50.
39. Rowlands AV, Pilgrim EL, Eston RG. Patterns of habitual activity across weekdays and weekend days in 9-11-year-old children. Prev Med. 2008;46(4):317-24. doi:10.1016/j.ypmed.2007.11.004.
40. Rush E, Coppinger T, Obolonkin V, Hinckson E, McGrath L, McLennan S et al. Use of pedometers to identify less active children and time spent in moderate to vigorous physical activity in the school setting. J Sci Med Sport. 2012;15(3):226-30. doi:10.1016/j.jsams.2011.11.001.
41. Sherar LB, Muhajarine N, Esliger DW, Baxter-Jones ADG. The relationship between girls' (8-14 years) physical activity and maternal education. Ann Hum Biol. 2009;36(5):573-83. doi:10.1080/03014460903071151.
42. Silva P, Rute S, Welk G, Mota J. Seasonal differences in physical activity and sedentary patterns: The relevance of the PA context. J Sports Sci Med. 2011;10(1):66-72.
43. Sirard JR, Kubik MY, Fulkerson JA, Arcan C. Objectively Measured Physical Activity in Urban Alternative High School Students. Med Sci Sports Exerc. 2008;40(12):2088-95. doi:10.1249/MSS.0b013e318182092b.
44. Soric M, Misigoj-Durakovic M. Physical activity levels and estimated energy expenditure in overweight and normal-weight 11-year-old children. Acta Paediatr. 2010;99(2):244-50. doi:10.1111/j.1651-2227.2009.01553.x.
45. Stone MR, Stevens D, Faulkner GEJ. Maintaining recommended sleep throughout the week is associated with increased physical activity in children. Prev Med. 2013;56(2):112-7. doi:10.1016/j.ypmed.2012.11.015.
46. Stone MR, Rowlands AV, Eston RG. Characteristics of the activity pattern in normal weight and overweight boys. Prev Med. 2009;49(2-3):205-8. doi:10.1016/j.ypmed.2009.06.012.
47. Sulemana H, Smolensky MH, Lai DJ. Relationship between physical activity and body mass index in adolescents. Med Sci Sports Exerc. 2006;38(6):1182-6. doi:10.1249/01.mss.0000222847.35004.a5.
48. Taylor RW, Farmer VL, Cameron SL, Meredith-Jones K, Williams SM, Mann JI. School playgrounds and physical activity policies as predictors of school and home time activity. Int J Behav Nutr Phys Act. 2011;8:38. doi:10.1186/1479-5868-8-38.
49. Treuth MS, Catellier DJ, Schmitz KH, Pate RR, Elder JP, McMurray RG et al. Weekend and weekday patterns of physical activity in overweight and normal-weight adolescent girls. Obesity. 2007;15(7):1782-8. doi:10.1038/oby.2007.212.

50. Trost SG, Pate RR, Freedson PS, Sallis JF, Taylor WC. Using objective physical activity measures with youth: How many days of monitoring are needed? Med Sci Sports Exerc. 2000;32(2):425-31. doi:10.1097/00005768-200002000-00025.

51. Uvacsek M, Toth M, Ridgers ND. Examining physical activity and inactivity in 9-12 years old children. Acta Physiol Hung. 2011;98(3):313-20. doi:10.1556/APhysiol.98.2011.3.8.

52. van Sluijs EMF, Fearne VA, Mattocks C, Riddoch C, Griffin SJ, Ness A. The contribution of active travel to children's physical activity levels: Cross-sectional results from the ALSPAC study. Prev Med. 2009;48(6):519-24. doi:10.1016/j.ypmed.2009.03.002.

53. Veitch J, Salmon J, Ball K. Individual, social and physical environmental correlates of children's active free-play: a cross-sectional study. Int J Behav Nutr Phys Act. 2010;7:11. doi:10.1186/1479-5868-7-11.

54. Wilkin TJ, Mallam KM, Metcalf BS, Jeffery AN, Voss LD. Variation in physical activity lies with the child, not his environment: Evidence for an 'activitystat' in young children (EarlyBird 16). Int J Obes. 2006;30(7):1050-5.
